# Impact of constipation on progression of Alzheimer's disease: A retrospective study

**DOI:** 10.1111/cns.13940

**Published:** 2022-08-08

**Authors:** Taizen Nakase, Yasuko Tatewaki, Benjamin Thyreau, Tatsushi Mutoh, Naoki Tomita, Shuzo Yamamoto, Yumi Takano, Michiho Muranaka, Yasuyuki Taki

**Affiliations:** ^1^ Smart Aging Research Center Tohoku University Sendai Japan; ^2^ Department of Aging Research and Geriatric Medicine, Institute of Development, Aging and Cancer Tohoku University Sendai Japan

**Keywords:** cognitive function, constipation, dementia, homocysteine, magnetic resonance imaging, worsening

## Abstract

**Background and Purpose:**

In terms of the gut‐brain axis, constipation has been considered to be an important factor of neurodegenerative diseases, although the exact mechanism is still controversial. Herein, we aimed to investigate the contribution of constipation to the progression of dementia in a retrospective study.

**Methods:**

Patients of Alzheimer's disease(AD) and amnestic mild cognitive impairment were consecutively screened between January 2015 and December 2020, and those of whom brain MRI and neuropsychological tests were performed twice were enrolled in this study. Participants were classified into with constipation (Cons[+], *n* = 20) and without constipation (Cons[−], *n* = 64) groups. Laboratory data at the first visit were used. Regression analysis was performed in MMSE, ADAS‐Cog, and the volumes of hippocampus on MRI‐MPRAGE images and deep white matter lesions (DWMLs) on MRI‐FLAIR images obtained at two different time points.

**Results:**

The main finding was that the Cons[+] group showed 2.7 times faster decline in cognitive impairment compared with the Cons[−] group, that is, the liner coefficients of ADAS‐Cog were 2.3544 points/year in the Cons[+] and 0.8592 points/year in the Cons[−] groups. Ancillary, changes of DWMLs showed significant correlation with the time span (*p* < 0.01), and the liner coefficients of DWMLs were 24.48 ml/year in the Cons[+] and 14.83 ml/year in the Cons[−] group, although annual rate of hippocampal atrophy was not different between the two groups. Moreover, serum homocysteine level at baseline was significantly higher in the Cons[+] group than Cons[−] group (14.6 ± 6.4 and 11.5 ± 4.2 nmol/ml, respectively: *p* = 0.03).

**Conclusion:**

There is a significant correlation between constipation and faster progression of AD symptoms along with expansion of DWMLs.

## Graphical abstract

Dementia patients with constipation showed higher serum homocysteine level and steeper cognitive decline along with faster expansion of brain white matter lesions than those without constipation. These findings suggest that chronic inflammatory response caused by constipation may lead to exacerbation of white matter lesions, resulting in the worsening of cognitive function.

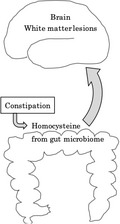

## 1 INTRODUCTION

Chronic constipation is frequently observed even in healthy adults, and its prevalence rate is increasing in elderly people.^1^, ^2^ Recently, the importance of association between constipation and alteration of gut microbiome has been paid attention to as a causative factor of the diseases.^3^, ^4^ In fact, altered composition of gut microbiome was reported in Alzheimer's disease (AD) patients.^5^ Altered gut microbiome has been revealed to be able to induce inflammatory response, leading to the damage of the central nervous system (CNS).^6^, ^7^ Moreover, it has been pointed out that metabolites as well as cytokines secreted from mucosal cells of the intestine were influenced by altered gut microbiome and affected the neuronal activity.^7^, ^8^ Especially, homocysteine has been reported to be a risk factor of cerebrovascular diseases.^9^, ^10^ Homocysteine is mainly produced from intestinal mucosal cells and can induce oxidative stress, causing the damage of vessel endothelium.^11^ On the contrary, several studies mentioned that mechanical or physiological factors are main cause of constipation.^12^, ^13^, ^14^ The severity of AD was reported to relate to constipation because of the decrease in water intake and mobility in AD patients.^13^ Thus far, although the gut–brain axis may play a critical role in the AD pathogenesis, actual mechanism is still under debate. This study aimed to (1) investigate whether constipation may associate with worsening of AD patients and (2) explore any factors which may relate to cognitive decline in AD patients with constipation.

## 2 METHODS

### 2.1 Patients

**FIGURE 1:**
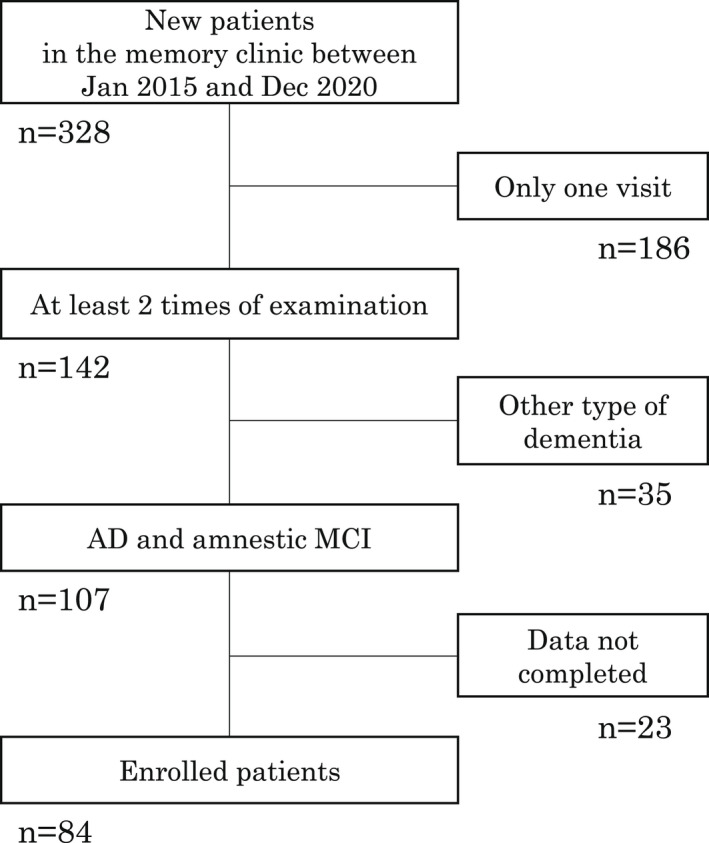
Flow chart of patients screening

New patients who visited our memory clinic between January 2015 and December 2020 were consecutively screened (*n* = 328) in a retrospective manner. Of those who were diagnosed AD or amnestic mild cognitive impairment (aMCI), patients who underwent brain magnetic resonance imaging (MRI) and neuropsychological tests at two time points (range 6–46 months) up to December 2021 were enrolled in this study (*n* = 84, 36 men and 48 women) (Figure 1). Inclusion criteria were (1) patients diagnosed with AD or aMCI, (2) performed at least two times of MRI and neuropsychological tests between the first visit and December 2021, (3) with complete information about past medical history, family history, medication record, lifestyle questionnaire, and blood test, and (4) do not consider prescribed medications. Exclusion criteria were (1) patients diagnosed with other type of dementia, (2) with incomplete clinical data, (3) could not perform MRI, and (4) did not have lifestyle questionnaire. Diagnosis of AD or aMCI was following the guidelines^15^, ^16^ and had been performed by registered neurologists or gerontologists. This diagnosis was obtained from patient's clinical record. We did not include the information about AD biomarkers, such as amyloid PET, CSF amyloid‐β42, and phosphorylated‐tau. Patients were asked about their condition of bowel movement at the first visit. Then, constipation was diagnosed following the guidelines.^17^, ^18^ Enrolled patients were classified into those with constipation (Cons[+] group, *n* = 20) and those without constipation (Cons[−] group, *n* = 64). Patient educational background, past medical history, presence of comorbidities (such as hypertension, hyperlipidemia, diabetes mellitus, and heart diseases) were extracted from the clinical records at the first visit. Laboratory data were obtained from blood examination at the first visit. Prescribed medications, including laxatives and antiflatulents, were obtained from medical record.

### 2.2 Cognitive assessment

Cognitive impairment was assessed using the results of the Japanese version of the Mini‐Mental State Examination (MMSE‐J) and Alzheimer's Disease Assessment Scale‐Cognitive Subscale Japanese version (ADAS‐Cog‐J). While the MMSE is used to screen cognitive dysfunction, because the ADAS‐Cog includes 11 tasks: Word recall, Naming objects and figures, Commands, Constructional praxis, Ideational praxis, Orientation, Word recognition, Language, Comprehension of spoken language, Word finding difficulty, and Remembering test instruction, we used it to assess the change in cognitive ability.^19^ The difference in the data between the first and second visits was defined as the cognitive decline.

### 2.3 Image processing

All the brain MR images were obtained using 3.0‐tesla MRI (Intera Achieva 3.0 T Quasar Dual) and 32‐element head coil. T1‐weighted 3D magnetization‐prepared rapid acquisition with gradient echo (MPRAGE) images were used for the analysis of hippocampal volume; [TR], 8.70 ms; echo time [TE], 3.1 ms; 8 degree flip angle; field of view [FOV], 256 × 256 × 180 mm; and voxel size, 0.7 × 0.7 × 0.7 mm^3^. Volumes of the region of interests were calculated by a computer software, FreeSurfer.^20^ The hippocampal volume was set as the sum of the right and left volume of hippocampus. Fluid‐attenuated inversion recovery (FLAIR) images were adopted for the analysis of the deep white matter lesions (DWMLs) and periventricular white matter lesions (PVWMLs). To obtain the volume of WMLs, the FLAIR images were processed using an automatic in‐house pipeline based on the Statistical Parametric Mapping ‐Lesion Segmentation Tool toolbox (https://www.applied‐statistics.de/lst.html)^21^ that segments the white matter hyperintensity. Images of the same subject at different time points were processed independently. The periventricular region was defined as a dilated (9 mm) mask of the ventricles, a subcortical region as a ribbon mask of the below gray matter, and an inner gray matter as the basal ganglia and thalamus, with the rest being labeled as deep white matter. From the lesion segmentation and region definition, we could then compute the total lesion volume in each brain region for all acquisitions of every subject.^22^ All volume measurements were automatically performed in a computer, and two researchers (YTat and MM) who were blinded from clinical data verified the accuracy.

### 2.4 Statistical analysis

Data are presented as mean ± standard deviation (SD) or as numbers and percentages. The clinical characteristics were compared between the Cons[+] group and the Cons[−] group by Mann–Whitney U‐test for mean variables since data were not in normal distribution, and by chi‐squared test for percentage variables. Temporal changes in MMSE‐J and ADAS‐Cog‐J were expressed as a relation between the duration of two time points and the difference in two scores. The temporal change amount of hippocampal volume and the temporal change rates of WMLs volume were presented as a relationship between these amounts and the duration of two time points. The change amount of hippocampal volume was calculated by subtracting the volume of the second examination from that of the first examination. The change rate of WMLs was calculated using the formula: (total volume of the second images – total volume of the first images) / total volume of the first images ×100%. Then, each data were dotted in a scatter graph according to the duration of two examinations. A linear regression model was adopted, and the correlation coefficient of the linear regression formula was evaluated as the worsening rate. Multivariate analysis was adopted for evaluating confounding factors, which showed *p* < 0.1 in the bivariate analysis by Mann–Whitney U‐test or chi‐squared test. For the multivariate analysis, the temporal change in ADAS‐Cog‐J score was transferred to the annual change in score by a formula: temporal change score / duration of two time point (years). The temporal change in DWML volume was transferred to the annual change rate by a formula: temporal change rate / duration of two time point (years). As confounding factors might not be in normal distribution, non‐parametric test was adopted for the multivariate analysis. Especially parameters in this study contains both continuous variables and nominal qualitative variables, Spearman's rank correlation coefficient test was used. Moreover, because this study contained fixed effects and random effects, the multivariate analysis was also performed by REML method as mixed effect model. For the calculation of sensitivity and specificity of constipation against cognitive worsening, ROC analysis was performed. JMP Pro15 software was used for the analysis. Statistical significance was set at *p* < 0.05.

### 2.5 Standard protocol approvals

All procedures in this study were approved by the Ethics Committee of Tohoku University School of Medicine (#2021‐1‐385). The institutional review board waived the need for patient consent, as this was a retrospective study, and all data were deidentified.

## 3 RESULTS

### 3.1 Patients' clinical characteristics

The baseline characteristics of all patients are shown in Table 1; the percentage of women was 57.1%, and the average age [SD] was 77.4 [6.5] years old. The number [%] of patients living alone was 8 [9.5%]. Around 24% of patients had been using care services. Constipation patients were included 23.8%. Patients diagnosed with AD was 45.2%, and all of them were taking anti‐dementia agents. Average [SD] length of follow‐up period was 17.4 [10.7] months. Anticholinergic drugs were prescribed to 19.0% of all patients. Regarding the treatment of constipation, 65.0% of patients with constipation had prescribed laxatives or antiflatulents.

**TABLE 1:** Patients' baseline characteristics

Total patient number	84
Women, *n* (%)	48 (57.1)
Age, ave. ± SD years	77.4 ± 6.5
Educational background, ave. ± SD years	13.1 ± 2.6
Past stroke history, *n* (%)	6 (7.1)
Hypertension, *n* (%)	54 (64.3)
Hyperlipidemia, *n* (%)	37 (44.0)
Diabetes mellitus, *n* (%)	21 (25.0)
Heart diseases, *n* (%)	6 (7.1)
Alcohol, *n* (%)	19 (22.6)
Smoking, *n* (%)	16 (19.0)
Constipation, *n* (%)	20 (23.8)
Care service use, *n* (%)	20 (23.8)
Living alone, *n* (%)	8 (9.5)
Diagnosis with AD, *n* (%)	38 (45.2)
MMSE‐J, ave. ± SD	24.7 ± 3.7
ADAS‐Cog‐J, ave. ± SD	11.8 ± 5.2
Follow‐up duration, ave. ± SD months	17.4 ± 10.7
Medications	
Calcium blocker, *n* (%)	34 (40.5)
ARB, *n* (%)	30 (35.7)
Statines, *n* (%)	29 (34.5)
Fibrates, *n* (%)	2 (2.4)
Antiplatelets, *n* (%)	17 (20.2)
Anticoagulation, *n* (%)	4 (4.8)
Anti‐dementia, *n* (%)	38 (45.2)
Antipsychotics, *n* (%)	22 (26.2)
Anticholinergic drugs (repost), *n* (%)	16 (19.0)
Laxatives, *n* (%)	14 (16.7)
Antiflatulents, *n* (%)	6 (7.1)

Abbreviations: ADAS‐Cog‐J, Alzheimer's disease assessment scale‐cognitive subscale Japanese version; ARB, angiotensin II receptor blocker; ave., average; MMSE‐J, mini‐mental state examination Japanese version; SD, standard deviation.

Then, comparing the two groups (Table 2), the percentage of women was not significantly different between the Cons[+] and Cons[−] groups. The average age at the first visit did not differ between the two groups. There were also no significant differences in the educational background, the frequency of hypertension, and diabetes mellitus between the two groups. The Cons[+] group showed significantly lower percentage of hyperlipidemia and significantly higher percentage of heart diseases compared with the Cons[−] group (25.0% vs. 50.0%: *p* = 0.0493 and 20.0% vs. 3.1%: *p* = 0.0105, respectively). Lifestyle backgrounds were almost the same between the two groups. The percentage of diagnosis with AD was also not significantly different between the two groups. Severity of cognitive impairment at the first visit was the same between the two groups, that is, the average scores of MMSE‐J and ADAS‐Cog‐J were not significantly different between the Cons[+] and Cons[−] groups (25.0 vs. 24.6 and 11.2 vs. 11.9, respectively).

**TABLE 2:** Background differences in each group at the first visit

	Cons (+)	Cons (−)	*p*
*N*	20	64	
Women (%)	45.0	60.9	0.2087
Age (ave. ± SD)	77.9 ± 6.7	77.0 ± 6.2	0.5677
Educational background (ave. ± SD, years)	12.8 ± 2.7	13.1 ± 2.6	0.8772
Hypertension (%)	75.0	60.9	0.2519
Hyperlipidemia (%)	25.0	50.0	0.0493
Diabetes mellitus (%)	40.0	20.3	0.0759
Heart diseases (%)	20.0	3.1	0.0105
Alcohol (%)	10.0	26.6	0.2187
Smoking (%)	5.0	23.44	0.1016
Care service use (%)	40.0	18.8	0.0713
Living alone (%)	10.0	9.38	1.0000
Diagnosis with AD (%)	60.0	40.6	0.1286
MMSE‐J (ave. ± SD)	25.0 ± 3.9	24.6 ± 3.7	0.5987
ADAS‐Cog‐J (ave. ± SD)	11.2 ± 5.1	11.9 ± 5.3	0.5674

Abbreviations: AD, Alzheimer's disease; ADAS‐Cog‐J, Alzheimer's disease assessment scale‐cognitive subscale Japanese version; Ave., average; MMSE‐J, Mini‐mental state examination Japanese version; SD, standard deviation.

### 3.2 Laboratory data

The laboratory data are presented in Table 3. Inflammatory factors, such as white blood cell count and C‐reactive protein levels, did not differ between the two groups. Although there was no difference in the amounts of vitamin B12 and folic acid, the serum homocysteine level was significantly higher in the Cons[+] group than in the Cons[−] group (average ± SD; 14.2 ± 6.8 and 11.5 ± 4.2 [nmol/ml]: *p* = 0.0463, respectively). Renal function, as assessed by estimated glomerular flow rate (eGFR), was within the normal range in both the Cons[+] and Cons[−] groups. There was no difference in the serum cholesterol levels between the two groups.

**TABLE 3:** Patients' laboratory data at the first visit

	Total	Cons (+)	Cons (−)	*p*
WBC (ave. ± SD, /μl)	6163.8 ± 1756.0	6489.5 ± 1736.8	6062.3 ± 1763.8	0.3578
CRP (ave. ± SD, mg/dl)	0.118 ± 0.184	0.124 ± 0.149	0.115 ± 0.195	0.8571
eGFR (ave. ± SD, ml/min/1.73m^2^)	69.2 ± 18.8	65.2 ± 21.8	70.4 ± 17.8	0.3045
TCho (ave. ± SD, mg/dl)	200.1 ± 38.1	197.5 ± 38.9	201.0 ± 38.1	0.7281
HDL (ave. ± SD, mg/dl)	61.6 ± 17.9	63.5 ± 20.4	60.9 ± 17.1	0.5886
LDL (ave. ± SD, mg/dl)	110.9 ± 29.1	108.4 ± 28.1	111.7 ± 29.7	0.6715
Homocysteine (ave. ± SD, nmol/ml)	12.1 ± 5.1	14.2 ± 6.8	11.5 ± 4.2	0.0463
Vitamin B12 (ave. ± SD, pg/ml)	565.8 ± 391.0	552.1 ± 416.9	569.9 ± 385.9	0.8753
Folic acid (ave. ± SD, mg/dl)	10.5 ± 5.2	9.5 ± 4.7	10.7 ± 5.3	0.3417

Abbreviations: CRP, C‐reactive protein; eGFR, estimated glomerular flow rate; HDL, high‐density lipoprotein; LDL, low‐density lipoprotein; SD, standard deviation; TCho, total cholesterol; WBC, white blood cell.

### 3.3 Cognitive decline

**FIGURE 2:**
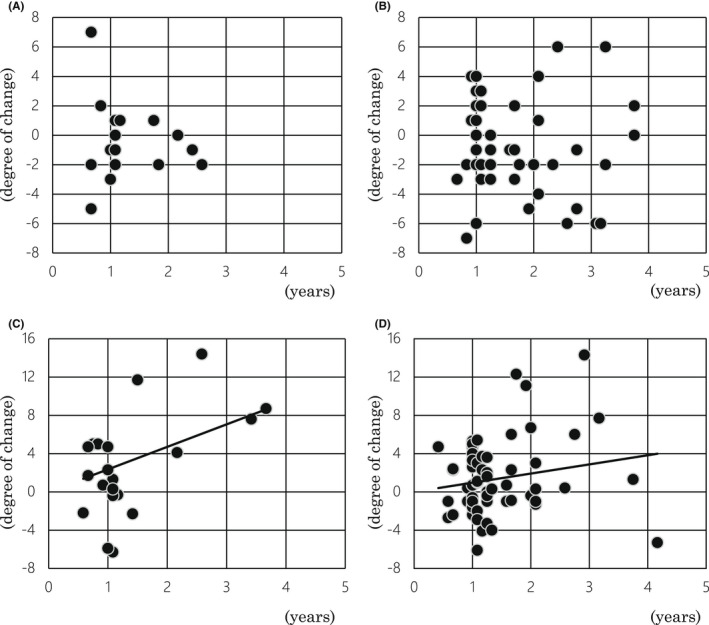
Scatter graphs of temporal change in MMSE‐J scores and ADAS‐Cog‐J scores. (A) There was no correlation between the temporal change in MMSE‐J score and the duration of two time points in the Cons[+] group. (B) The Cons[−] group also showed no correlation between the temporal change in MMSE‐J score and the duration of two time points. (C) The Cons[+] group showed a significant correlation between the temporal change in ADAS‐Cog‐J score and the duration of two time points. The solid line indicates the regression line: y = 0.1962x, *R* ^2^ = 0.2697, *p* = 0.0012. (D) The Cons[−] group also showed a significant correlation between the temporal change in ADAS‐Cog‐J score and the duration of two time points. The solid line indicates the regression line: y = 0.0716x, *R* ^2^ = 0.0423, *p* = 0.0044. The X‐axis shows the duration between the first and second examinations (years). The Y‐axis shows the change in score.

Cognitive alterations were expressed by means of the temporal change in the MMSE‐J and ADAS‐Cog‐J scores (Figure 2). Two cases in the Cons[+] group and 10 cases in the Cons[−] group were lost in MMSE‐J data. According to the linear regression model, there was no significant correlation between the temporal change in MMSE‐J score and the duration of two time points (years) in both the Cons[+] and Cons[−] groups. Significant correlations were observed between the temporal change in ADAS‐Cog‐J score and the duration of two time points (years) in both the Cons[+] and Cons[−] groups (y = 0.1962x, *R* ^2^ = 0.2697: *p* = 0.0012 and y = 0.0716x, *R* ^2^ = 0.0423: *p* = 0.0044, respectively). In other words, the Cons[+] group showed a 2.74 times faster decline in cognitive function than the Cons[−] group.

### 3.4 Alteration of Brain Lesions

**FIGURE 3:**
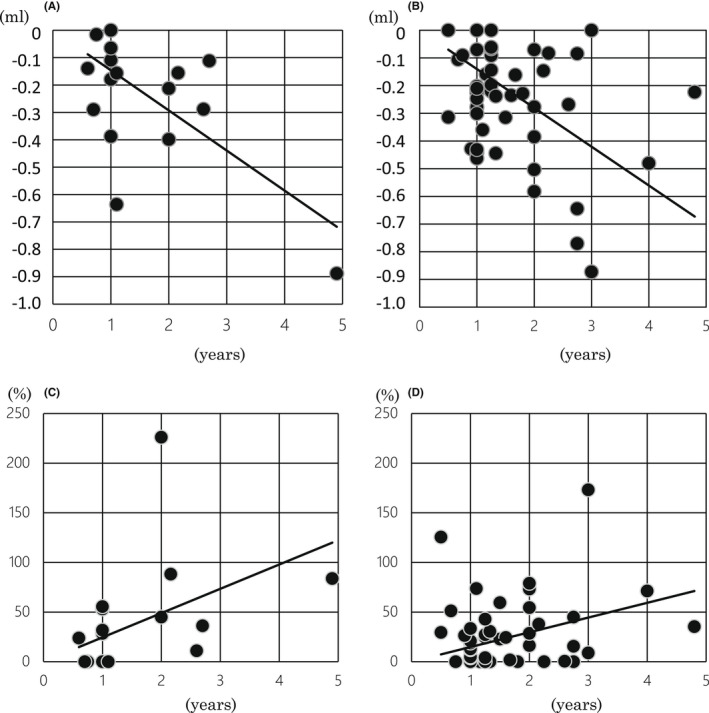
Scatter graphs of temporal change amount of hippocampus (A and B) and temporal change rate of DWML volume (C and D). Both the Cons[+] and Cons[−] groups showed a significant correlation between the temporal change amount and the duration of two time points (A and B, respectively). The solid lines represent the regression lines: y = −0.146x, *R* ^2^ = 0.3869, *p* < 0.001 in the Cons[+] group and y = −0.140x, *R* ^2^ = 0.013, *p* < 0.001 in the Cons[−] group. (C) The Cons[+] group presented a significant correlation between the temporal change rate of DWML volume and the duration of two time points. The solid line indicates the regression line: y = 24.48x, *R* ^2^ = 0.1329, *p* = 0.0019. (D) The Cons[−] group also presented a significant correlation between the temporal change rate of DWML volume and the duration of two time points. The solid line indicates the regression line: y = 14.83x, *R* ^2^ = 0.0329, *p* < 0.001. The X‐axis represents the duration between the first and second examinations (years). The Y‐axis shows the change rate (%).

Sixty‐seven patients (79.8%) were examined using brain MRI at two time points during the observation period (*n* = 17 in Cons[+] and *n* = 50 in Cons[−] groups). There was no significant difference of hippocampal volume at the first examination between the two groups (average ± SD [mL]; 4.44 ± 0.97 in Cons[+] and 4.82 ± 0.85 in Cons[−]: *p* = 0.2171). Moreover, the annual volume reduction in hippocampus was almost the same between the two groups (correlation coefficient: −0.146 ml/year, *R* ^2^ = 0.3869: *p* < 0.0001 in Cons[+] and − 0.140 ml/year, *R* ^2^ = 0.013: *p* < 0.0001 in Cons[−]) by means of the regression analysis (Figure 3A,B).

Regarding the DWML and PVWML volumes at the first examination, both data were not significantly different between the Cons[+] and Cons[−] groups (average ± SD; 4.40 ± 6.81 and 4.64 ± 6.84 [ml]: *p* = 0.6862, 12.00 ± 9.42 and 11.21 ± 9.01 [mL]: *p* = 0.6337, respectively). By the regression analysis (Figure 3C,D), significant correlations were observed between the temporal change rates of DWML and the duration of two time points (years) in both the Cons[+] and Cons[−] groups (y = 24.48x, *R* ^2^ = 0.1329: *p* = 0.0019 and y = 14.83x, *R* ^2^ = 0.0329: *p* < 0.001). According to the correlation coefficient of the formula, the expansion of the DWML was 1.65 times faster in the Cons[+] group than in the Cons[−] group.

The annual volume change in PVWML did not differ between the two groups (data not shown).

### 3.5 Multivariate analysis

We used confounding factors, which showed the difference between the Cons[+] and Cons[−] groups with *p* < 0.1 by the bivariate analysis. That is, hyperlipidemia, diabetes mellitus, heart diseases, and serum homocysteine level were adopted for comparing with the annual change in ADAS‐Cog‐J score and the annual change rate of DWML volume in the multivariate analysis, Spearman's rank correlation coefficient test (Table 4). Only constipation presented the significant correlation with the annual change in ADAS‐Cog‐J score (*p* = 0.0288) and the annual change rate of DWML volume (*p* = 0.0395), while mixed effect model by means of REML method showed no significant correlation of constipation with the confounding factors (Table 5).

**TABLE 4:** Correlation coefficient for Spearman's rank correlation coefficient test

	ADAS‐Cog‐J	*p*	DWML	*p*
Constipation	0.2387	0.0288	0.2252	0.0395
Hyperlipidemia	−0.0653	0.5551	−0.0921	0.4047
Diabetes mellitus	−0.0170	0.8779	0.0434	0.6952
Heart diseases	0.0172	0.8769	0.0480	0.6647
Serum homocysteine	0.0247	0.8332	0.0390	0.7400

Abbreviations: ADAS‐Cog‐J, Alzheimer's disease assessment scale‐cognitive subscale Japanese version; DWML, deep white matter lesion.

**TABLE 5:** Correlation coefficient for REML method of mixed effect model

	ADAS‐Cog‐J	*p*	DWML	*p*
Constipation	0.1857	0.0908	0.1689	0.1245
Hyperlipidemia	−0.0384	0.7287	−0.0237	0.8304
Diabetes mellitus	−0.0285	0.7970	0.0263	0.8124
Heart diseases	0.0437	0.6932	0.0497	0.6532
Serum homocysteine	0.0715	0.5419	−0.0896	0.4445

Abbreviations: ADAS‐Cog‐J, Alzheimer's disease assessment scale‐cognitive subscale Japanese version; DWML, deep white matter lesion.

### 3.6 Impact of existing constipation

ROC analysis was performed for evaluating the sensitivity and specificity of constipation against the exacerbation of cognitive decline. When the cognitive worsening was set at the annual worsening rate of ADAS‐Cog‐J higher than 4.1 points/year, maximum AUC (0.60385) was obtained. Then, the sensitivity and specificity of the existence of constipation pointed at 45.0% and 81.5%, respectively.

## 4 DISCUSSION

Our results clearly showed that constipation significantly correlated with a steeper decline in cognitive function and greater increase in DWML volume in patients with AD and aMCI. Moreover, higher level of serum homocysteine was observed in those with constipation.

As a primary analysis, patients with constipation experienced cognitive decline at a rate over twice that experienced by patients without constipation. Constipation is frequently observed even in healthy adults, and its prevalence rate increases in the elderly.^1^, ^2^ Moreover, constipation can be a risk of neurodegenerative diseases, such as Parkinson's disease (PD) and Dementia with Lewy body (DLB),^23^, ^24^ while association of constipation with AD pathology is controversial.^13^ Recently, the association between constipation and the gut microbiome has been extensively studied. It was reported that constipation could trigger structural changes in the microbiome,^25^, ^26^ and conversely, alteration of microbiome was reported to induce constipation.^4^ In fact, an altered gut flora was reported in AD patients compared with control counterparts.^5^, ^27^, ^28^ The altered intestinal flora was reported to damage on the activity of the central nervous system through an inflammatory response by stimulating intestinal immune cells and vagus nerve.^6^, ^7^ Moreover, it has been pointed out that metabolites as well as cytokines secreted from mucosal cells of the intestine were influenced by altered gut microbiome and affected the neuronal activity.^7^, ^8^ Although we did not observe gut microbiota, high serum homocysteine level was related to steeper cognitive decline in patients with constipation. Interestingly, altered gut microbiome, which was observed in PD patients, was reported to contribute to the increase in homocysteine level.^29^ Moreover, because homocysteine is produced in the metabolic process of levodopa, pathological impact of homocysteine has been investigated in PD patients. Serum homocysteine level was significantly high in PD patients, and that level was correlated with brain atrophy.^30^ Higher homocysteine level correlated with larger size of the third ventricle and less volume of mesencephalon.^31^ Cognitive decline in PD patients was reported to show higher homocysteine level, lower folate and vitamin B12 levels, although it is unveiled the role of homocysteine in the cognitive decline.^32^ Homocysteine has been reported to be a risk factor for cardiovascular and neurodegenerative diseases, acting by several mechanisms. Oxygen radicals might be produced through the cascade of homocysteine metabolism causing endothelial cell damage,^10^ and homocysteine could activate matrix metaroproteinase‐9, a membrane disrupting agent, leading to a direct influence on the cell membrane.^9^ Of great interest, about 25% of systemic circulating homocysteine is produced by intestinal mucosal cells.^11^ Therefore, it can be said that neuronal damage might be indirectly accelerated by constipation, leading to the worsening of cognitive impairment.

As a secondary analysis, a faster increase in DWML volume, which has been reported as a cause of cognitive decline in AD patients,^33^, ^34^ was observed in patients with constipation. Regarding DWML pathogenesis, decreased cerebral blood flow or a blood–brain barrier disruption induced by endothelial damage of cerebral deep small arteries have been reported.^33^, ^34^, ^35^ Experimental studies have revealed that chronic cerebral hypoperfusion causes myelin sheath damage and neuronal death,^36^, ^37^ while endothelial cells of deep small arteries have been reported to be injured by chronic inflammatory response caused by hypertension, diabetes mellitus, dyslipidemia, smoking, and chronic kidney disease.^38^ With respect to our study, no difference was observed in the incidence of hypertension and diabetes mellitus between the Cons[+] and Cons[−] groups, and the incidence of hyperlipidemia was rather lower in the Cons[+] group. Therefore, instead of vascular risk factors, involvement of inflammatory response may be expected in expansion of DWMLs in patients with constipation. In our findings, the reduction in hippocampal volume was not different between patients with and without constipation. It can be said that, although hippocampus is critical part of AD pathology, cerebral white matter is also important for maintaining cognitive function in AD patients. In fact, default mode network (DMN) which is affected by white matter lesions plays a critical role in AD pathology.^39^ Rectified DMN was reported to be an imaging marker which will distinguish AD from DLB.^40^

According to a multivariate analysis by means of correlation coefficient test, only constipation presented the significant contribution to the cognitive decline assessed by ADAS‐Cog‐J and the DWML expansion. On the contrary, a mixed effect model revealed no significant association of constipation with any of confounding factors. Moreover, a result of ROC analysis showed that sensitivity was 45.0% and specificity was 81.5%, when the cognitive worsening was defined as ADAS‐Cog‐J score ≧4.1 point/year. Therefore, it can be said that constipation may not be a necessary condition for steeper cognitive decline. Rather, a condition without constipation may support a relatively slow decline in cognitive impairment. Meanwhile, according to a recent report in which the effect of hyper‐homocysteine was restricted in mice with apolipoprotein E (ApoE) ε4 allele and such effect showed sex differences,^41^ our results in multi variate analysis might be partially explained by that ApoE polymorphism and sex difference were not considered in this study.

This study has several limitations. First, the analyzed data were from a single institution, and the number of enrolled cases was relatively small. We therefore need to confirm our findings in a larger study. However, previous studies have reported that the prevalence rate of constipation was 15%–20% in a population over 60 years old and 25% in patients over 70 years old.^13^ In this study, the prevalence rate of constipation was 23.8%, which suggests that our cases could partially represent patients of interest. Second, we could not evaluate the influence of anti‐constipation therapy because this was a retrospective study. However, we may say that current anti‐constipation therapy was not enough for restraining cognitive decline, as 65% of patients with constipation in this study were prescribed anti‐constipation agents. Third, even though the average age, educational background, and lifestyle backgrounds were similar between patients with and without constipation, we did not assess daily physical activity which might influence constipation. The amount of physical activity should be explored in future analysis. Fourth, disease duration might influence on the cognitive decline; however, the onset age of AD patients could not be obtained from clinical records. While because there were no significant differences of MMSE‐J, ADAS‐Cog‐J scores, average age, and percentage of diagnosis with AD at the first examination between the two groups, it can be said that backgrounds of cognitive impairment were not different between patients with and without constipation at the first visit. Fifth, patients who were performed two times of clinical examinations were enrolled in this study. In our memory clinic, as a regional center for dementia, most of diagnosed patients are repeatedly followed. However, there is a possibility in which only intended patients were included and some patients were lost in follow‐up.

Nevertheless, it can be said that reduction in serum homocysteine level may have a potential for treatment of AD patients, and this kind of intervention will need to be introduced in early stage or prodromal stage of disease. For example, ApoE ε4 carriers were reported to show altered cerebral blood flow even in middle‐aged participants.^42^ The effect of ketone diet on the AD mice model was reported to reduce microglial activation and neuroinflammation which were observed only in cases of longer treatment group and younger initiated group.^43^ Future studies need to be conducted to explore the details of our findings.

In conclusion, there is a significant correlation between constipation and an exacerbation of cognitive decline in AD and amnestic MCI patients along with DWML expansion. As the statistical power of this study was weak, a larger prospective study must be considered for confirming our observations in the future.

## AUTHOR CONTRIBUTIONS

Taizen Nakase involved in conceptualization, data curation, formal analysis, investigation, methodology, project administration, and writing—original draft. Yasuko Tatewaki involved in data curation, formal analysis, investigation, methodology, project administration, and writing—original draft. Benjamin Thyreau involved in data curation, formal analysis, investigation, methodology, and writing—original draft. Tatsushi Mutoh involved in conceptualization, investigation, methodology, and writing—review and editing. Naoki Tomita involved in methodology, and writing—review and editing. Shuzo Yamamoto involved in writing‐review and editing. Yumi Takano involved in investigation, writing‐review and editing. Michiho Muranaka involved in writing—review and editing. Yasuyuki Taki involved in investigation, project administration, and writing—review and editing.

## CONFLICT OF INTEREST

None.

## Data Availability

All relevant data are presented in this article. Original data are available from the corresponding author on reasonable request. This study followed the Strengthening the Reporting of Observational Studies in Epidemiology (STROBE) statement.
